# Cricoarytenoid joint arthritis: a possible complication of dermatomyositis

**DOI:** 10.11604/pamj.2020.36.74.18891

**Published:** 2020-06-09

**Authors:** Chadi Farah, Ouidade Aitisha Tabesh, Jad Okais, Arlette Hajjar, Amine Haddad

**Affiliations:** 1 Otolaryngology - Head and Neck Surgery Department, Hotel Dieu de France University Hospital, Saint Joseph University, School of Medicine, Alfred Naccache Street, Achrafieh, Beirut, Lebanon,; 2 Rheumatology Department, Hotel Dieu de France University Hospital, Saint Joseph University, School of Medicine, Beirut, Alfred Naccache Street, Achrafieh, Beirut, Lebanon,; 3 Rheumatology Department, Mount Lebanon Hospital, Camille Chamoun Street, Hazmieh, Beirut, Lebanon

**Keywords:** Cricoarytenoid joint arthritis, dermatomyositis, larynx, steroid therapy

## Abstract

Cricoarytenoid joint arthritis is most frequently reported in Rheumatoid Arthritis and in other systemic diseases such as Sjogren’s syndrome, Systemic Lupus Erythematosus, Ankylosing Arthritis, Juvenile Chronic Arthritis, and autoimmune hepatitis but it has not been reported in dermatomyositis. In this paper, we report the case of a 43 years-old woman treated for dermatomyositis who presented with hoarseness and severe odynophagia. The laryngoscopy revealed the presence of an extensive white swelling of the left cricoarytenoid joint with reduced mobility of the left vocal cord, consistent with left cricoarytenoid joint arthritis, which has not previously been described in dermatomyositis to our knowledge. Treatment with high doses of prednisone produced a complete resolution of the laryngeal symptoms.

## Introduction

Cricoarytenoid joint arthritis (CAJA) is an entity that is manifested clinically by hoarseness, and pain on talking and swallowing. An inflammatory process that could be isolated as in infection, or associated to other systemic signs and symptoms, causes this entity. CAJA is described in the literature as part of Rheumatoid Arthritis, Sjogren’s syndrome, Systemic Lupus Erythematosus, Ankylosing Arthritis, Juvenile Chronic Arthritis, and autoimmune hepatitis [1-10]. CAJA could be sometimes mistaken for a laryngeal mass of neoplastic origin [9], and can cause severe airway obstruction if not treated in its early stage. In this paper, we present a case of a patient diagnosed as having dermatomyositis (DM) presenting a CAJA misdiagnosed as a laryngeal tumor. To our knowledge this is the first reported case of CAJA in a DM patient.

## Patient and observation

A 43 years-old non-smoker woman diagnosed as having DM due to a weakness of the proximal muscles and a characteristic heliotropic rash. Investigations showed a high level of creatine phosphokinase, a myogenic pattern with spontaneous fibrillation and an early recruitment of the muscles of the four limbs on the electromyography (EMG) and the muscle biopsy revealed inflammatory infiltrates compatible with DM. Her disease was uncontrolled with Prednisone 20 mg/day, Hydroxychloroquine 400mg/day and Methotrexate 7.5 mg/week.

Later on, she presented hoarseness, mild dyspnea, dysphagia, and odynophagia associated with a referred left otalgia, but without aspirations. A laryngoscopy done elsewhere showed a left glottic mass and a surgical treatment was planned, but the patient presented to our department for a second opinion. This patient had frequent flare-ups of her underlying disease and wasn’t compliant to her treatment and follow-up. At presentation, she had no fever, no infectious signs and symptoms and no stridor.

**Figure 1:**
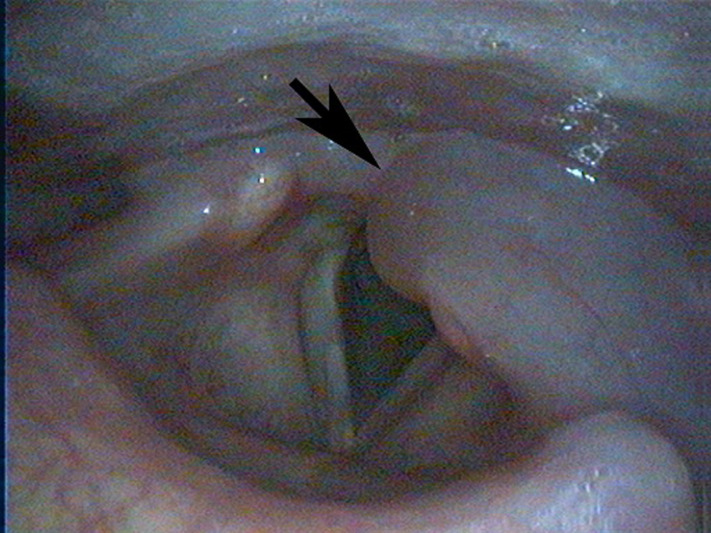
laryngoscopy showing the left cricoarytenoid joint arthritis

**Figure 2:**
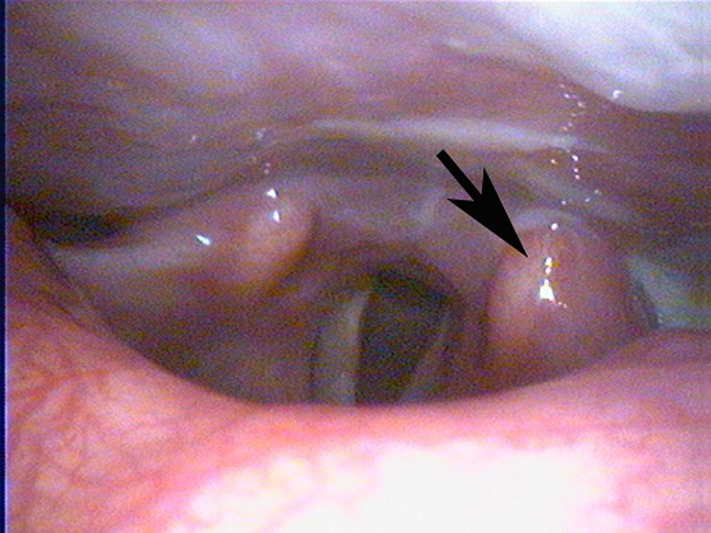
laryngoscopy showing the resolution of the left cricoarytenoid joint arthritis after treatment with steroids

A flexible laryngoscopy done in our department showed a left cricoarytenoid white swelling with no mucosal lesions but with a reduced mobility of the left vocal cord (Figure 1). After increasing the dose of prednisone to 60mg/day, the flexible laryngoscopy done 9 days later showed a disappearance of the mass (Figure 2) with the resolution of the laryngeal and extra-laryngeal symptoms. The diagnosis of CAJA was considered.

## Discussion

DM is an autoimmune myopathy characterized clinically by proximal muscle weakness, muscle inflammation, characteristic rash and, frequently, the presence of autoantibodies [11]. Diagnosis of definite DM requires the presence of characteristic rash as well as at least three of the four signs of muscle inflammation and weakness; symmetric proximal weakness, elevated levels of muscle enzymes (creatine kinase, aspartate aminotransferase, lactate dehydrogenase and aldolase), electromyographical changes consistent with irritable myopathy, or necrosis and inflammation on muscle biopsy [11]. Our patient fulfilled the criteria of DM. Our patient presented with laryngeal symptoms of hoarseness, mild dyspnea, dysphagia, and odynophagia associated with a referred left otalgia. Laryngoscopy showed edema, with white swelling of the left CAJ and a reduced mobility of the left vocal cord. There were no neurological deficits and no signs of infection. The treatment with steroids improved the inflammatory symptoms of DM as well as the mobility of the vocal cords and the laryngeal symptoms. The rapid response to steroids and the complete resolution of the laryngeal signs and symptoms confirms the diagnosis of an inflammatory process in the CAJ. In the absence of signs of infection and the presence of an autoimmune disease, the diagnosis of CAJA related to DM can be considered in this patient. According to the published literature, as in our patient, CAJA is manifested clinically by hoarseness, and pain on talking and swallowing. Other symptoms that should alert one to the possibility of CAJA include foreign body sensation in the throat, dyspnea and pain radiating to one or both ears. Upper airway obstruction with stridor is relatively uncommon but should imply an urgent airway management [9, 10, 12]. With regard to diagnosis, low-voltage radiographs, computed tomography (CT) and magnetic resonance imaging (MRI) are useful for detecting upper airway obstruction, if present, and the diagnosis of CAJA can be confirmed by laryngoscopy, as in our case. Laryngoscopy reveals red or white swelling of the arytenoids in the acute phase, edema of the vocal folds in the chronic phase and thickened mucosa over the arytenoids in both phases [3].

Treatment of CAJA largely depends on the presence of airway obstruction. In the acute phase, airway management is the most important consideration because hypoxia can cause irreversible brain damage and be life-threatening leading to death if treatment was delayed [3, 5]. In the presence of airway obstruction, intubation is difficult and can damage arytenoids especially in patients with bilateral involvement and thus can delay the establishment of airway patency before the onset of severe dyspnea. For these reasons, tracheostomy is the preferred treatment in patients with stridor and bilateral midline arytenoid fixation. In addition to airway management, it has been reported that the administration of steroids and epinephrine inhalation is effective for treating CAJA [3]. Of major importance, fundamental and definitive therapy for the flare-up of the underlying disease should be successively provided as well as an adequate maintenance treatment to prevent further flare-ups. If systemic therapy fails, corticosteroid injection of the CAJ is advocated [9]. In our patient, dyspnea was mild and CAJA was unilateral as seen on laryngoscopy. Treatment with steroids was sufficient to produce a complete resolution of laryngeal signs and symptoms and a maintenance treatment was successful in providing a good control of her disease.

CAJA is reported in the literature as caused by an infectious process or part of an autoimmune and inflammatory disease and can be the only laryngeal manifestation of the underlying disease. CAJA was most frequently reported in Rheumatoid Arthritis [3, 5, 9, 10], with a prevalence that varies between 32-75% on laryngoscopy [9]. CAJA is also found in Sjogren’s syndrome [2], Ankylosing Spondylitis [4], Systemic Lupus Erythematosus [5-7], Juvenile Chronic Arthritis [8], and autoimmune hepatitis [1]. To our knowledge, no case of CAJA in a patient with DM was reported in the literature. As in our case, CAJA can be sometimes misdiagnosed as a laryngeal tumor. Chen *et al* [9], reported a case of a patient with RA and a CAJA that was presumed to be an aggressive squamous cell carcinoma (SCC). Laryngoscopy was used to differentiate SCC from CAJA. In addition to showing the distinctive features of CAJA, laryngoscopy is used to distinguish between mucosal and submucosal lesions, as submucosal lesions are much less likely to be SCC. Furthermore, SCC of the larynx is more invasive and often presents with central necrosis. If suspected on laryngoscopy and clear distinction can’t be made, CT scan can be employed to analyze the mass and define its extent as well as the presence of invasive features in order to differentiate between SCC and CAJA [9].

It is important to differentiate between a CAJA and SCC of the larynx due to the difference in treatment and prognosis. In our case, laryngoscopy showed characteristic features of CAJA with no mucosal lesion, which ruled out the possibility of having an invasive laryngeal tumor. In our patient, with the absence of infectious signs, the low suspicion of a laryngeal tumor, the evidence of DM and the complete resolution of signs and symptoms with steroids, we consider that the laryngeal manifestation was primarily caused by a CAJA as a complication of DM. CAJA can sometimes lead to death when causing airway obstruction [5]. The CAJ is not readily accessible for inspection or palpation during routine examination, and symptoms are often unnoticed by the patient until advanced stages of CAJ inflammation [5]. Knowing that CAJA is confirmed by laryngoscopy, and laryngoscopy specialists are not always available in Emergency Departments, physicians should be aware of this complication in the case of DM to avoid severe consequences and treat the patient immediately [3].

## Conclusion

In this paper, we presented the first case of CAJA to be reported in a patient with DM. CAJA is a condition that should be kept in mind as it can be life-threatening causing stridor and airway obstruction. Therefore, if detected, it should be diagnosed and treated immediately. When a patient with a history of an autoimmune or inflammatory disease presents with a submucosal mass on laryngoscopy, CAJA should be strongly considered. Treatment should first assure airway patency, then steroids should be used to reduce inflammation and maintenance treatment should be given in order to reduce flare-ups of the underlying disease.
